# Design and synthesis of novel dihydropyridine- and benzylideneimine-based tyrosinase inhibitors

**DOI:** 10.3389/fphar.2024.1332184

**Published:** 2024-03-26

**Authors:** Ifraz Ahmad, Warda Parveen, Shah Noor, Zahoor Udin, Amjad Ali, Ijaz Ali, Riaz Ullah, Hamid Ali

**Affiliations:** ^1^ Chemistry Department, The University of Lahore, Punjab, Pakistan; ^2^ Key Laboratory of Automobile Materials, Department of Material Sciences and Engineering, Jilin University, Changchun, China; ^3^ Chemistry Department, Gomal University, Dera Ismail Khan, Pakistan; ^4^ Faculty of Biological Sciences, Department of Biochemistry, Quaid-i-Azam University, Islamabad, Pakistan; ^5^ Centre for Applied Mathematics and Bioinformatics (CAMB), Gulf University for Science and Technology, Hawally, Kuwait; ^6^ Department of Pharmacognosy, College of Pharmacy King Saud University, Riyadh, Saudi Arabia; ^7^ Department of Biosciences, COMSATS University Islamabad, Islamabad, Pakistan

**Keywords:** tyrosinase inhibitors, tyrosine kinase, skin care, antibrowning, anticancer, amlodipine, dihydropyridine, benzylideneimine

## Abstract

Tyrosinase (TYR) inhibitors are very significant as they inhibit enzyme tyrosinase activity, and its inhibition is vital for skin care, anticancer medication, and antibrowning of fruits and vegetables. This work presents a novel and economical route for the preparation of new synthetic tyrosinase inhibitors using amlodipine **(4)**. The novel conjugates **6 (a–o)** were designed, synthesized, and characterized by spectroscopic analyses, including Fourier transform infrared and low- and high-resolution mass spectroscopy. The purified compound **4** was refluxed with various aldehydes and ketones **5 (a–o)** for 5–8 h in methanol at 60°C–90°C. This research modified the drug in a step-by-step manner to develop therapeutic properties as a tyrosinase inhibitor. The structures of synthesized ligands **6 (a–o)** were established based on spectral and analytical data. The synthesized compounds **6 (a–o)** were screened against tyrosinase enzyme. Kojic acid was taken as standard. All the prepared compounds **6 (a–o)** have good inhibition potential against the enzyme tyrosinase. Compounds **6o**, **6b**, **6f**, and **6k** depicted excellent antityrosinase activity. Compound **6k**, with an IC_50_ value of 5.34 ± 0.58 µM, is as potent as the standard kojic acid (IC_50_ 6.04 ± 0.11 µM), standing out among all synthesized compounds **6 (a–o)**. The *in silico* studies of the conjugates **6 (a–o)** were evaluated via *PatchDock*. Compound **6k** showed a binding affinity score of 8,999 and an atomic contact energy (ACE) value of −219.66 kcal/mol. The structure–activity relationship illustrated that the presence of dihydropyridine nuclei and some activating groups at the ortho and para positions of the benzylideneimine moiety is the main factor for good tyrosinase activity. The compound **6k** could be used as a lead compound for drug modification as a tyrosinase inhibitor for skin care, anticancer medication, and antibrowning for fruits and vegetables.

## 1 Introduction

Tyrosinase (TYR), a binuclear copper-containing enzyme belonging to the oxidoreductase class of enzymes that catalyze oxidation, is a primary target for the treatment of over-pigmentation disorder and associated skin problems because it directly contributes to the synthesis of melanin (Roulier et al., 2020). Protein tyrosine kinase (PTK) is one of the many cellular proteins that have been identified as an anticancer study target as a result of the quick advancement of life science research (e.g., DNA, tubulin, and essential enzymes) (Mongre et al., 2021). PTK is a group of tyrosine kinase-active proteins that contribute to the control of several physiological and biochemical processes, including cell growth, differentiation, and death (Wang et al., 2019). PTK expression may be abnormal, which may promote tumor neovascularization, invasion, metastasis, and resistance to therapy (Drake et al., 2014; Knosel et al., 2014). As a result, PTK has evolved into an important target for antitumor drug research (Jiao et al., 2018). One of the biggest issues facing food companies is enzymatic browning, particularly for fruits, vegetables, and seafood items. Enzymatic and nonenzymatic oxidation are the two processes that cause browning. Producing poisonous and antinutritional substances could further diminish the nutritional value and safety of food (Atasever et al., 2013; Aydin et al., 2015; Arnold and Gramza-Michalowska, 2022). Tyrosinase enzymatic oxidation of phenols, which results in the degradation of plant-based foods and beverages, can cause degradation of important amino acids, inhibits proteolytic and glycolytic enzymes, and, therefore, reduces digestibility and nutrition and releases hazardous chemicals (Qian et al., 2020). Tyrosinase and its polyphenolic substrates combine with oxygen to cause the enzymatic browning of fruits, vegetables, and beverages after brushing, peeling, and crushing operations that cause cell structures to break down (Moon et al., 2020). This enzyme occurs naturally in a wide variety of organisms, including bacteria, fungi, higher plants (including mushrooms, bananas, apples, pears, potatoes, avocados, and peaches), and animals (Yuan et al., 2019). Tyrosinase and phenolic substrate concentrations, oxygen availability, pH, temperature, and other factors all affect how quickly an object will turn brown through enzymatic means (Panis and Rompel, 2022). Food additives like reducing agents and enzyme inhibitors can be used to reduce browning.

Hydroquinone-based tyrosinase inhibitors utilized for skin disorders are linked to a range of unfavorable effects, including contact dermatitis, irritation, transitory erythema, burning, prickling feeling, leukoderma, chestnut patches on the nails, hypochromia, and ochronosis, and they are potentially carcinogenic to mammalian cells (Bilovol et al., 2021). The natural substance arbutin, a prodrug of hydroquinone, decreases or prevents melanin formation by blocking tyrosinase (Boo, 2021). Though chemically unstable, natural forms of arbutin have the ability to produce hydroquinone, which is catabolized into benzene metabolites, which may be hazardous to bone marrow (Zhou et al., 2013). Due to its cancer-causing properties and instability during storage, the use of kojic acid in cosmetics has been restricted (Masub and Khachemoune, 2020). The degradation of L-ascorbic acid is rapid and sensitive to heat (Yin et al., 2022). Ellagic acid is insoluble and, therefore, poorly bioavailable (Arulmozhi et al., 2013), and the melanogenic path remains undecided for tranexamic acid (Tse and Hui, 2013).

In brief, tyrosinase inhibitors, besides being used to treat specific dermatological conditions linked to melanin hyperpigmentation, play a vital role in the cosmetics industry due to their ability to brighten skin and reduce sunburn-related pigmentation. Tyrosinase inhibitors also find applications in the food and beverage industry and as anticancer medication. Developing effective tyrosinase inhibitors is important in order to address these concerns. We have synthesized *de novo* conjugates of amlodipine that have a dihydropyridine-type heterocyclic ring structure and a benzylideneimine moiety, which are very effective as tyrosinase inhibitors, via facile and routine methods.

## 2 Materials and methods

The novel compounds **6 (a–o)** were designed rationally via *in silico* studies and synthesized, characterized, and evaluated for their tyrosinase inhibition potential.

### 2.1 Design

Tyrosinase (EC 1.14.18.1), also known as polyphenol oxidase, is a type of multi-functional binuclear copper ion metalloenzyme that is abundantly present in animals, plants, and microbes. It is crucial for several physiological and pathological functions (Casanola-Martin et al., 2014). The primary enzyme in the manufacture of melanin is TYR. A number of pigmented skin illnesses, including malignant melanoma, can develop as a result of high levels of TYR activity that result in melanin buildup (Buitrago et al., 2016).

**SCHEME 1:**
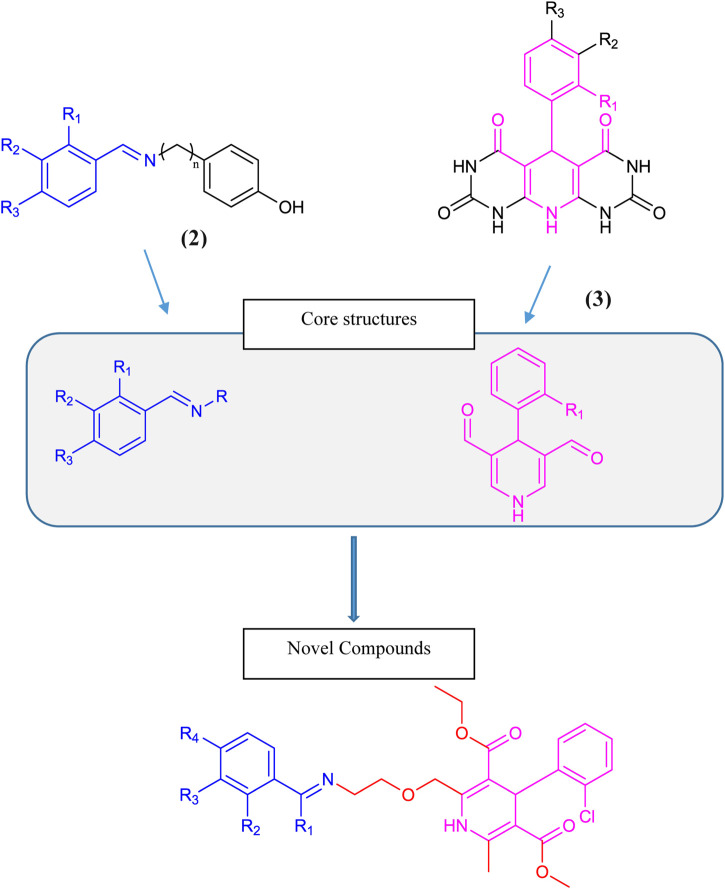
Rational design of conjugates **6 (a–o)**.

Dihydropyridine-based tyrosinase inhibitors were reported by (Bhosle et al., 2018), as given in Scheme 1 structure **(3)**. Amlodipine **(4),** which is commercially available in the form of amlodipine besylate **(1)** with dihydropyridine nuclei, is a commonly prescribed medication for hypertension and angina. It belongs to a class of drugs called calcium channel blockers, which work by relaxing and widening blood vessels to improve blood flow and reduce cardiac workload. Amlodipine **(4)** is known for its long-lasting effects, with a half-life of approximately 30 h, making it convenient for once-daily dosing. It is a well-tolerated drug with few side effects, making it a reliable choice for patients with cardiovascular diseases (Tang et al., 2016). Overall, amlodipine plays a significant role in managing hypertension and improving heart health (Zhou et al., 2022). Over the past 2 decades, it has remained under study for several types of biological activities. Based on its active dihydropyridine structure, we analyzed it for the design of novel biologically effective tyrosinase inhibitors.

Benzylideneimine-type tyrosinase inhibitors were reported by Wang et al. (2019) as given in structure (2) in Scheme 1. Bae et al. (2012) and Lima et al. (2013) also described benzylideneimine-containing tyrosinase inhibitors. In this research, we synthesized novel tyrosinase inhibitors by improving amlodipine chemically based on the proposition that dihydropyridine and benzylideneimine play a role in antityrosinase activity. Hence, the benzylideneimine moiety with different substituents on the benzyl ring has been introduced to the original structure of amlodipine via chemical reactions. In this way, 15 novel compounds were synthesized. The rational designing of these novel amlodipine conjugates **6 (a–o)** as tyrosinase inhibitors is given below in Scheme 1.

### 2.2 *In silico* studies

The crystal structure of the tyrosinase protein was retrieved from the Research Collaboratory for Structural Bioinformatics (RCSB) Protein Data Bank (PDB ID 2y9x). The coordinate files were subjected to Discovery Studio 4.5 Visualizer for pre-docking receptor preparation by removing water molecules and adding hydrogen atoms. Ligands **6 (a–o)** were docked with tyrosinase (2y9x) by *PatchDock*. *PatchDock* is a molecular docking tool aimed at finding docking transformations that generate good molecular shape complementarity based on shape complementarity principles (Schneidman-Duhovny et al., 2005). The input files comprise the receptor protein and ligand in PDB format.

The *PatchDock* server offers multiple solutions. “Solution 1” was selected as it surrounded the most significant residues as a binding pocket for docking analyses assigned in the crystal structure of the tyrosinase receptor (2y9x) (Silva et al., 2017; Nazir et al., 2022). The docked structures were analyzed *via* Discovery Studio 4.5 Visualizer.

**FIGURE 1:**
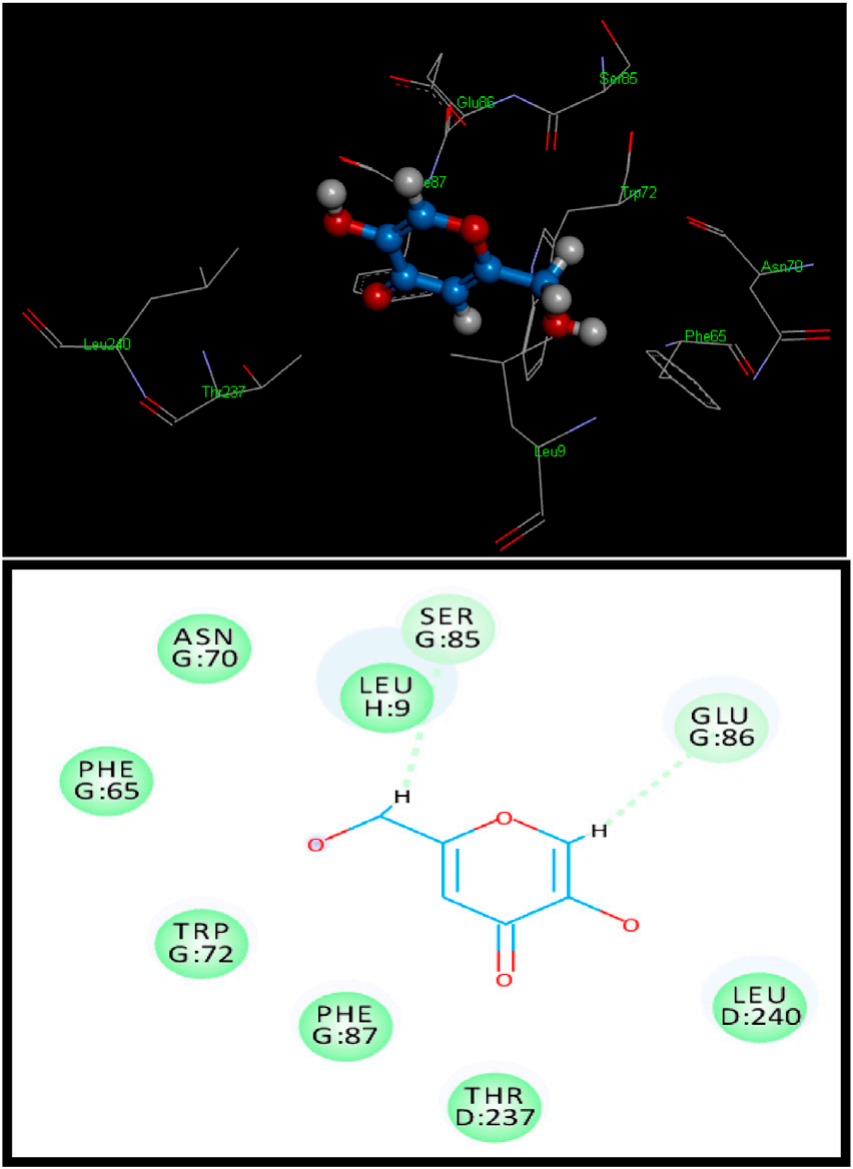
3D (above**)** and 2D (below) binding site contacts of standard kojic acid with a tyrosinase binding pocket.

**FIGURE 2:**
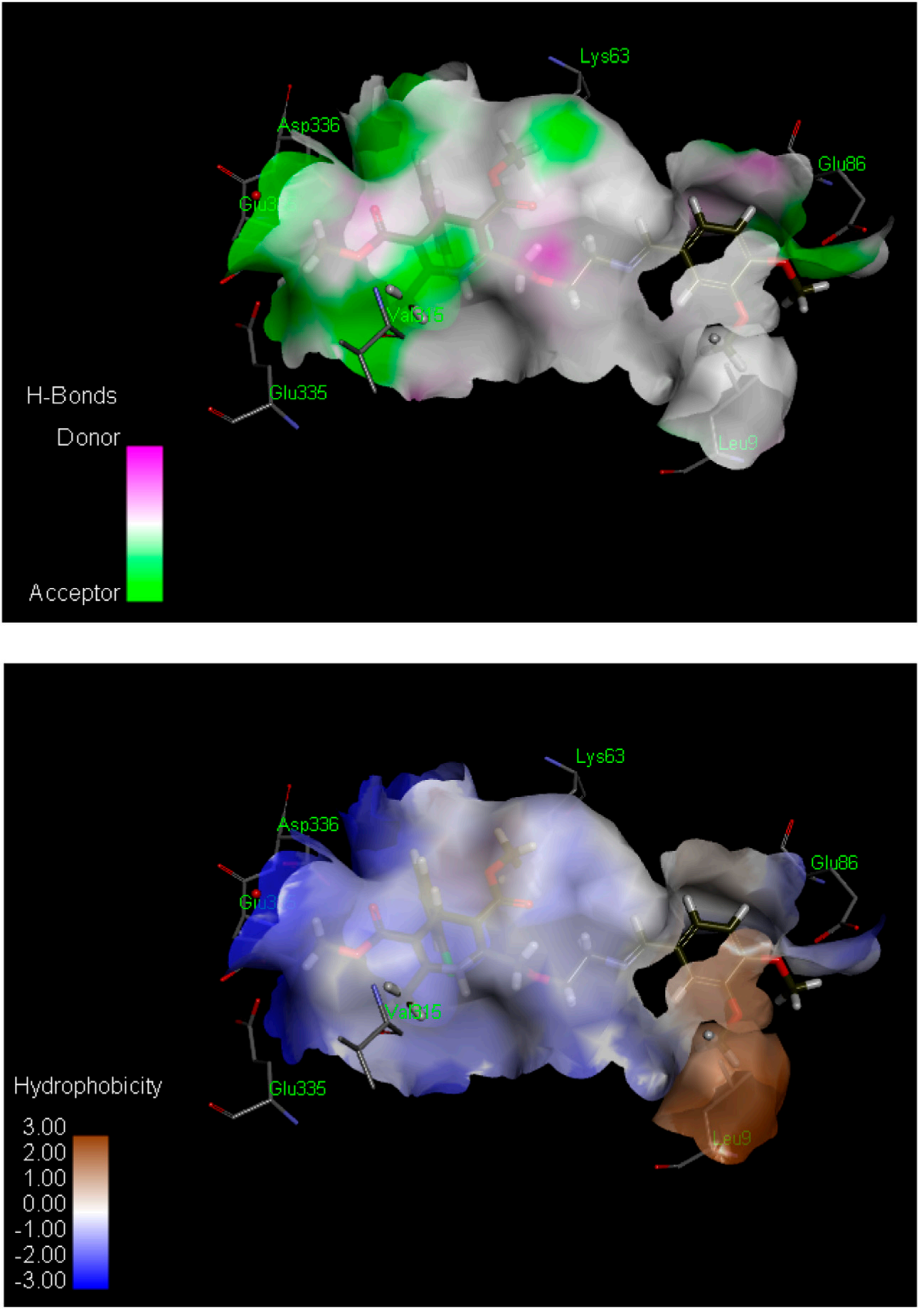
Hydrogen bond (above) and hydrophobic interaction (below) analyses of the most active inhibitor, **6k**. The structural elements (atoms and torsions) that contribute favorably to the overall binding energy are colored blue, those that contribute unfavorably are colored pink, and neutral elements are colored white.

**FIGURE 3:**
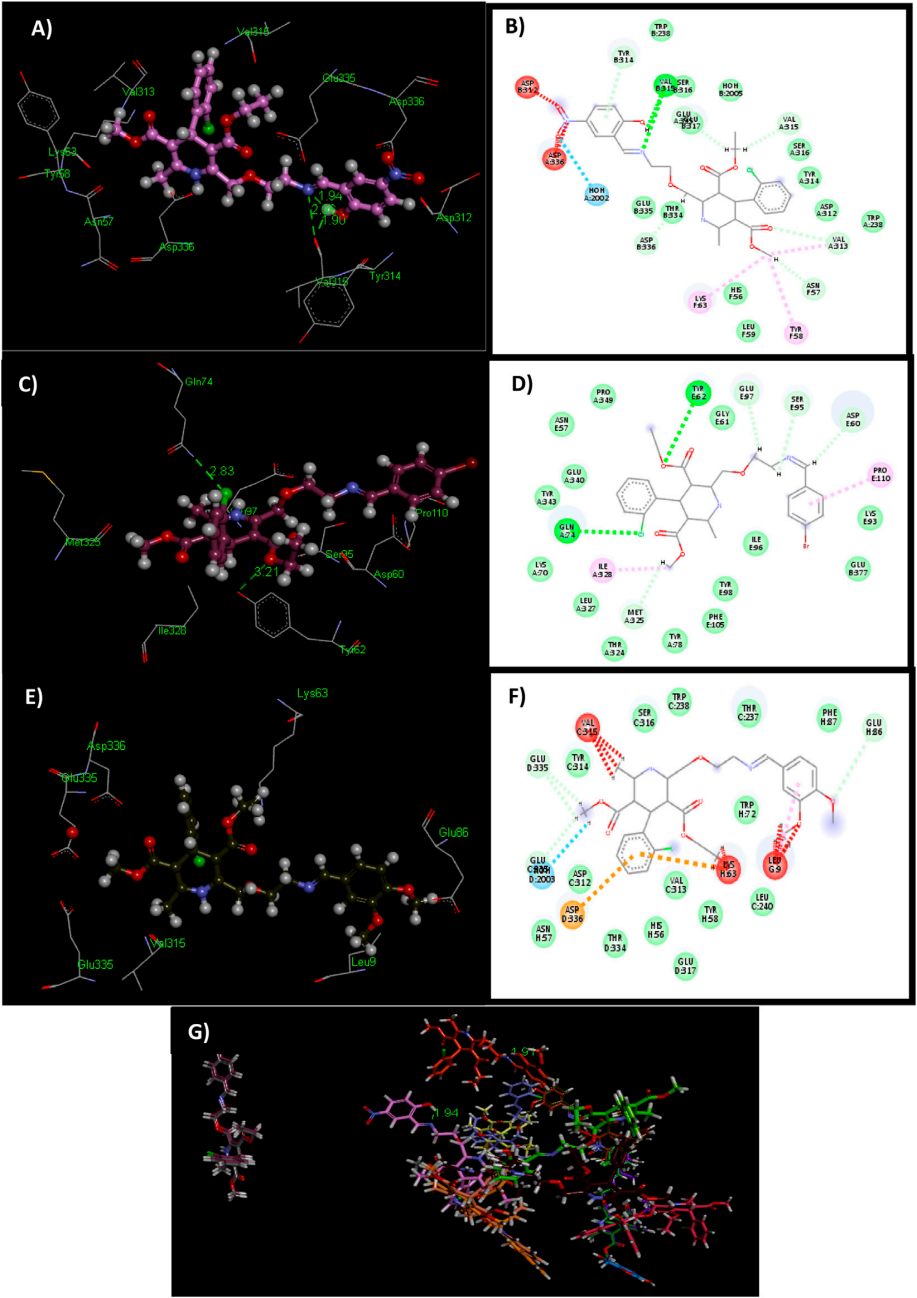
3D and 2D structures of docked ligands **6o (A, B)**, **6f (C, D)**, and **6k (E, F)**. Illustrating unfavorable protrusion (red), H–bond (light green), pi–alkyl (light pink), pi–cation (brown), and amide pi–stack (pink) interactions within a binding pocket of the tyrosinase protein (PDB ID: 2y9x). **(G)** Overlap of bound conformations of kojic acid (blue) with ligands **6d** (gray), **6a** (yellow), **6e** (mauve), **6o** (pale pink), **6n** (green), **6i** (brown), **6f** (magenta), **6k** (olive green), **6c** (mustard)**, 6m** (purple), **6h** (pink), **6g** (bright green), **6h** (beige), **6j** (orange), and **6b** (chocolate brown).

The binding affinity scores and ACEs of the docked ligand complexes were assessed. The hydrogen bonding and hydrophobic contacts of each ligand were evaluated within the binding pocket of the receptor protein. The conformations of the ligands that demonstrated the highest biological activities are explained in Supplementary Table S1 and Figures 1–3, which show their favorable interactions in the binding pouches. We examined the docked complexes of ligands **(6 (a–o)** and kojic acid to qualitatively estimate their biological activities (IC_50_) and identify the molecular basis for these effects.

### 2.3 Chemistry

All of the chemicals and solvents were purchased from Sigma-Aldrich and Alfa Aesar and utilized directly for experimentation. The melting points of the compounds were determined by using the Gallenkamp melting point apparatus via the open capillary method. Fourier transform infrared (FTIR) spectra were recorded on a Bruker alpha 2 FTIR spectrophotometer with a spectral range of 4,000–400 cm^−1^. Low-resolution mass spectra (LRMS) were obtained using an ESI LC TOF 6224 (Agilent) under the positive ion mode. High-resolution mass spectra (HSMS) were obtained on a 9.4 T Bruker FT-ICR-MS spectrometer in MeOH: MeCN (1: 1). Thin-layer chromatography (TLC) was determined on silica gel plates (Analtech 02521), using the solvent systems EtOAc:hexanes (8:2) and CHCl_3_:CH_3_OH (1:1).

#### 2.3.1 Purification of amlodipine **(4)**

Amlodipine besylate **(1)** was used as the starting material. It was then processed to obtain a purified form **4** by mixing it with a combination of dichloromethane and water in a 1:1 ratio. Following this step, the mixture underwent basification at a pH of 11. Subsequently, an extraction process using dichloromethane as the solvent was conducted. The final purification of the extracted substance was achieved through preparative TLC. The purified extract was dried and analyzed for physical and chemical characterization.

#### 2.3.2 Synthesis of compounds **6 (a–o)**

**SCHEME 2:**
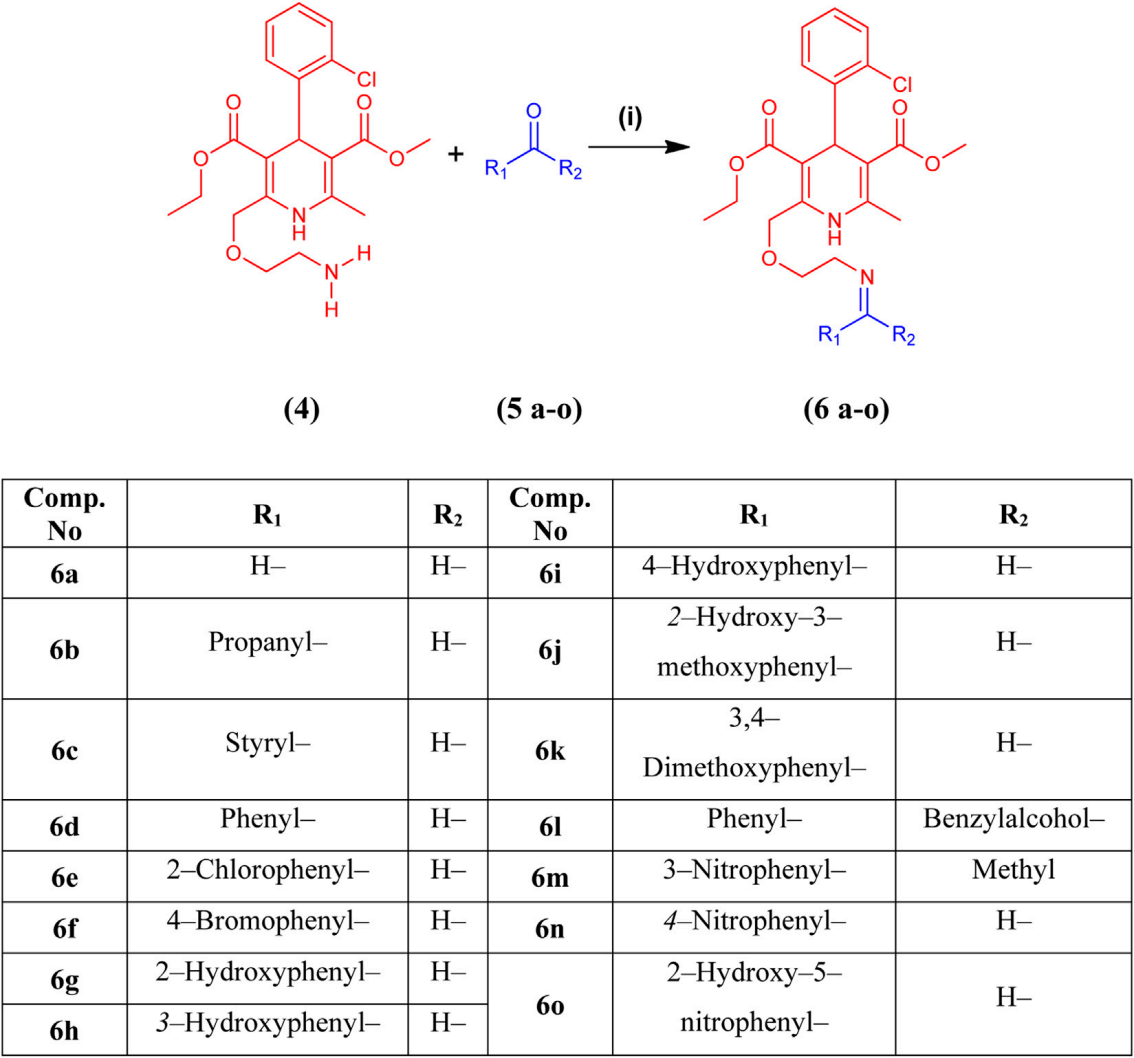
Synthesis of amlodipine derivatives **6 (a–o)** from amlodipine **(4)** and aldehydes or ketones **5 (a–o)**. Reagents and conditions: **(i**) CH_3_CH_2_OH and CH_3_COOH, reflux for 5–8 h at 60°C–90°C.

Fifteen novel amlodipine conjugates **6 (a–o)** were synthesized by the conventional imine formation reaction. All the compounds were synthesized by the one-pot reaction of various aldehydes and ketones **5 (a–o)** with compound **4** in ethanol under reflux. Amlodipine **(4)** and substituted aldehydes and ketones **5(a–o)** were refluxed using ethanol and a catalytic amount of CH_3_COOH for approximately 5–8 h. Imines were obtained as sole products (Scheme 2). The completion of the reaction was observed by TLC. The solvent system was chloroform and methanol in a ratio of 9.9:0.1. Then, the reaction mixture was quenched by aqueous NaHCO_3_ solution (1 M). Next, the organic product was extracted using DCM (3 × 10 mL). Anhydrous MgSO_4_ was used to dry the combined organic extract. Later, the solid product was filtered. After evaporation, preparative TLC was used for purification. Chloroform and methanol (90:10) were used as a solvent system. The purified organic product was dried over silica gel beads in desiccators for a week. Finally, the dried compound was collected for further analysis (Mocanu and Cernătescu, 2008). Characterization of synthesized compounds **6(a–o)** was done via FTIR, high-resolution mass spectroscopy (HRMS), and LRMS.

### 2.4 Biological evaluation for tyrosinase inhibition

Tyrosinase assay materials (100 μL) were mixed with 100 mM phosphate buffer (60 μL), pH 6.8, 10 μL (5 units) of mushroom tyrosinase enzyme, and 10 μL (0.5 mM) of the test substance (Lee, 2002) in a 96-well-plate. The assay contents were pre-incubated for 5 min at 37°C. Then, 20 μL of L-dopamine (10 mM) was added as a substrate, the contents were mixed, and the solution was incubated for a period of 30 min. The absorbance was recorded at 490 nm by using a Synergy HT BioTek 96-well plate reader.

The enzyme inhibition (%) was determined by the given formula

Inhibition (%) = 100 − (Abs of the test sample/Abs of control × 100)

The IC_50_ values (the concentration of a drug or inhibitor needed to inhibit a biological process or response by 50%) of compounds were calculated using EZ-Fit Enzyme Kinetics Software (Perrella Scientific Inc. Amherst, USA) (Asadzadeh et al., 2016; Shao et al., 2018; Wang et al., 2019).

## 3 Results and discussion

### 3.1 Molecular docking of tyrosinase inhibitors

To assess the tyrosinase inhibition potential, compounds **6 (a–o)** and standard kojic acid were docked against the tyrosinase enzyme via the software, as explained above. To obtain a qualitative estimation and to identify the molecular origin of the analyzed biological activities (IC_50_), the docked complexes of ligands (**6 (a–o)** and **kojic acid**) were investigated. The initial assessment of the docked complexes of tyrosinase **(6 (a–o)** and **kojic acid)** disclosed that **6k** and **6o** have superior docking scores than the standard, kojic acid.

Standard kojic acid was also docked with tyrosinase for comparison and had a score of 2,530 with an ACE value of −68.92 kcal/mol (Figure 1). Kojic acid has shown hydrogen bonding with Tyr^128^. The length of the hydrogen bond was 3.35 Å, and hydrophobic contact potential with pocket amino acids Leu^9^, Asn^70^, Phe^65^, Trp^72^, Phe^87^, Thr^237^, and Leu^240^ was identified (Supplementary Table S1).

The ligand **6k** showed the highest IC_50_ value (Figure 2; Table 1) and exhibited excellent binding affinity with a score of 8,999 and an ACE value of −219.66 kcal/mol. No hydrogen bond was observed between **6k** and the binding pocket (PDB ID 2y9x). Visual examination of the ligand **6k** depicted the potential for hydrophobic interactions with pocket amino acid residues Ser^316^, Trp^238^, Thr^237^, Phe^37^, Trp^72^, Leu^240^, Tyr^58^, Val^313^, Glu^317^, His^56^, Thr^334^, Asn^57^, Asp^312^, and Tyr^314^. These interactions can contribute significantly to comparatively superior binding affinity. A pi–alkyl interaction can also be visualized between Leu^9^ and the 3-hydroxy-4-methoxyphenyl species (Supplementary Table S1).

**TABLE 1:** Compounds **(a-o)** having different substituents with respect to R1 and R2 shown below.

Comp. no.	R_1_	R_2_	Comp. no.	R_1_	R_2_
**a**	H–	H–	**i**	4-Hydroxyphenyl–	H–
**b**	Propanyl–	H–	**j**	*2-*Hydroxy-3-methoxyphenyl–	H–
**c**	Styryl–	H–	**k**	3,4-Dimethoxyphenyl–	H–
**d**	Phenyl–	H–	**l**	Phenyl–	Benzylalcohol–
**e**	2-Chlorophenyl–	H–	**m**	3-Nitrophenyl–	Methyl
**f**	4-Bromophenyl–	H–	**n**	*4-*Nitrophenyl–	H–
**g**	2-Hydroxyphenyl–	H–	**o**	2-Hydroxy-5-nitrophenyl–	H–
**h**	*3-*Hydroxyphenyl–	H–			

Study of the contacts between ligand **6o** and the tyrosinase pocket discloses the binding affinity as a score of 8,908 and an ACE of −152.73 kcal/mol. Ligand **6o** exhibited two hydrogen bond interactions among the carbonyl group of amino acid Val^315^ and the –C=N (1.90 Å) and –OH (2.96 Å) groups of ligand **6o**. However, ligand **6o** showed hydrophobic interaction with Trp^238^, Ser^316^, Ser^316^, Glu^317^, HOH ^2005^, Tyr^314^, Leu^59^, Trp^238^, Asp^312^, His^56^, Thr^334^, and Glu^335^ amino acid residues. The pi–alkyl interaction can also be visualized between Tyr^58^ and the methyl formate directly attached to the dihydropyridine ring moiety (Supplementary Table S1).

Similarly, ligand **6f** and the tyrosinase pocket had a binding affinity score of 8,758 and an ACE value of −146.96 kcal/mol. Two hydrogen bonds were observed between the amino group directly attached to the carbonyl of Gln^74^ and the 2-chlorophenyl group (2.83 Å). Another hydrogen bond was observed between the 4-hydroxyphenyl group of Tyr^62^ and the oxygen of the methyl formate moiety (3.21 Å). In contrast, ligand **6f** depicted hydrophobic contact potential with Tyr^343^, Glu^340^, Asn^57^, Pro^349^, Gly^61^, Lys^93^, Ile^96^, Glu^377^, Tyr^98^, Phe^105^, Tyr^78^, Thr^324^, Leu^327^, and Lys^70^ amino acid residues. The pi–alkyl interaction can also be visualized between Pro^110^ and the 4-hydroxyphenyl moiety (Supplementary Table S1).

### 3.2 Chemical characterization

The structures of the title compounds **6 (a–o)** were confirmed by FTIR, LRMS, and HRMS, which revealed that the results were in accordance with the proposed and calculated values and testify to our synthetic approach. The overall results are given in Supplementary Material Section 2.

### 3.3 Tyrosinase inhibition activity

A series of novel amlodipine conjugates (**6 a–o**) were analyzed for their tyrosinase inhibition activity. All the compounds were found to be active against the enzyme tyrosinase. The strength of the tyrosinase inhibition activity of compounds **6 (a–o)** decreases as follows: **6k > 6o > 6b > 6f > 6e > 6c > 6n > 6a > 6g > 6l > 6h > 6d > 6j > 6i > 6m** (Table 2).

**TABLE 2:** Determination of IC_50_ values of tyrosinase inhibition activity of the amlodipine conjugates (**6a–o**).

Comp. no.	-R_1_	-R_2_	Conc. (mM)	Tyrosinase inhibition activity	
				% inhibition	IC_50_^a^ (µM)
**6a**	H-	H-	0.5	91.32 ± 0.89	10.28 ± 0.68
**6b**	Propanyl-	H-	0.5	94.67 ± 0.86	7.22 ± 0.78
**6c**	Styryl-	H-	0.5	92.92 ± 0.89	9.11 ± 0.45
**6d**	Phenyl-	H-	0.5	90.68 ± 0.87	15.14 ± 0.79
**6e**	2-Chlorophenyl-	H-	0.5	91.78 ± 0.67	8.11 ± 0.85
**6f**	4-Bromophenyl-	H-	0.5	94.47 ± 0.56	7.97 ± 0.68
**6g**	2-Hydroxyphenyl-	H-	0.5	90.33 ± 0.88	11.74 ± 0.45
**6h**	*3-*Hydroxyphenyl-	H-	0.5	90.87 ± 0.98	13.42 ± 0.88
**6i**	4-Hydroxyphenyl-	H-	0.5	90.88 ± 0.98	15.84 ± 0.35
**6j**	*2-*Hydroxy-3-methoxyphenyl-	H-	0.5	88.63 ± 0.97	15.44 ± 0.65
**6k**	3,4-Dimethoxyphenyl-	H-	0.5	95.18 ± 0.69	5.34 ± 0.58
**6l**	Phenyl-	Benzyl alcohol-	0.5	90.47 ± 0.87	13.36 ± 0.95
**6m**	3-Nitrophenyl-	Methyl	0.5	89.72 ± 0.76	16.41 ± 0.45
**6n**	*4-*Nitrophenyl-	H-	0.5	90.45 ± 0.98	9.21 ± 0.55
**6o**	2-Hydroxy-5-nitrophenyl-	H-	0.5	93.22 ± 0.77	6.03 ± 0.95
**Kojic acid**	Std		0.5	93.50 ± 0.91	6.04 ± 0.11

Results are the mean of three independent experiments (*n* = 3) ± SD.

The results revealed that compound **6k** is the most potent compound of series **6 (a–o),** having an IC_50_ of 5.34 ± 0.58 µM, which is as potent as the standard kojic acid, with an IC_50_ of 6.04 ± 0.11 µM. Three other members of series **6 (a–o)** also showed good inhibition; for example, **6o** had an IC_50_ of 6.03 ± 0.95 µM, **6b** had an IC_50_ of 7.22 ± 0.78 µM, and **6f** had an IC_50_ of 7.97 ± 0.68 µM.

**SCHEME 3:**
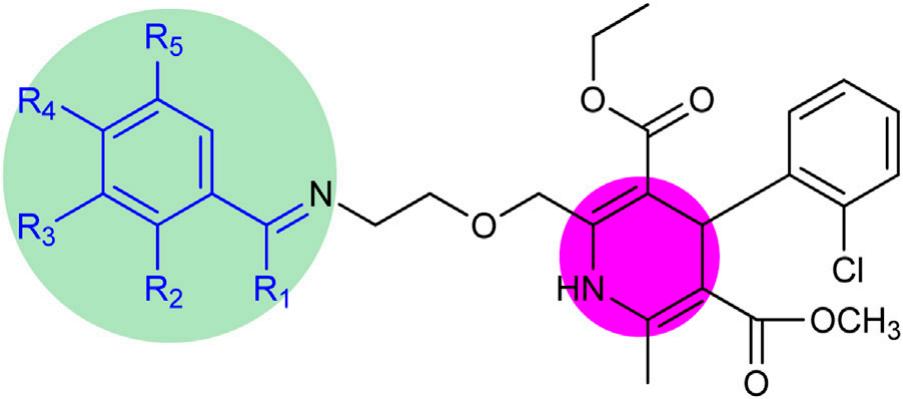
Core structure of compounds **6 (a–o)** showing the benzylideneimine group in the green-shaded region and dihydropyridine in the pink. R_1_ = H, CH_3_ and R_2_, R_3_, R_4_, R_5_ = H, CH_3_O, CH_3_CH_2_O, OH, NO_2_, Br, Cl.

The structure–activity relationship revealed two main core structures in the synthesized compounds **6 (a–o)** that are responsible for antityrosinase activity, the pyridine ring and the benzylideneimine moiety (Scheme 3). The dihydropyridine nuclei in the molecule are the main cause of the antityrosinase activity (Bhosle et al., 2018). Therefore, **6b** (IC_50_ 7.22 ± 0.78 µM) has the third highest value of tyrosinase inhibition among compounds **6 (a–o)** due to the presence of two dihydropyridine rings. Electron-withdrawing substituents of the benzylideneimine moiety of compounds **6 (d–o)** contribute to the change in the antityrosinase activity. The electron-donating substituents at the ortho or para positions or the electron-withdrawing groups at the meta position enhance the tyrosinase inhibition activity. It is noteworthy that the substituent position and number on the benzylideneimine aromatic ring of compounds **6 (d–o)** are important for inhibition activity, as the hydroxyl group at the ortho position enhances antityrosinase activity (Lima et al., 2013). A methoxy substituent at the para position of benzylideneimine is very important to enhance the tyrosinase inhibition activity (Bae et al., 2012). Hence, compound **6k** gives the best value of tyrosinase inhibition among **6 (a–o)**. The order of activity for ortho-, meta-, and para-hydroxy substituted benzylideneimine is *o*–OH > *p*–OH > *m*–OH, as given by the compounds **6g** (IC_50_ 11.74 ± 0.45 µM) **> 6h** (IC_50_ 15.84 ± 0.35 µM) **>** (**6i** IC_50_ 13.42 ± 0.88 µM). Among the nitro-substituted benzylideneimine, the para nitros have a lower IC_50_ than the meta nitro-substituted, as **6n** (IC_50_ 9.21 ± 0.55 µM) has a smaller value of tyrosinase inhibition than **6o** (6.03 ± 0.95 µM). Non-substituted benzylideneimine-containing compounds give poor inhibition, as shown by compounds **6d** (IC_50_ 15.14 ± 0.79 µM) and **6l** (IC_50_ 13.36 ± 0.95 µM). However, it is difficult to establish the SAR of the conjugates **6 (a–o),** probably due to multiple factors, as size, shape, electronegativity, and polarizability of the ligand play a role in enzyme inhibition.

## 4 Conclusion

A series of novel amlodipine conjugates **6 (a–o)** were designed, synthesized, and characterized by spectroscopic analyses, including FTIR, LRMS, and HRMS. The purified ligand **4** was refluxed with various aldehydes and ketones **6 (a–o)** for 5–8 h in ethanol at 60°C–90°C. The research work was progressively completed so that the drug could be altered to develop therapeutic properties as a tyrosinase inhibitor. The structures of the prepared derivatives **6 (a–o)** were established on the basis of analytical and spectral data. The synthesized conjugates **6 (a–o)** were evaluated for antityrosinase activity against mushroom tyrosinase enzyme, for which kojic acid was taken as standard. The biological activity assay revealed that all the compounds have inhibition potential against the enzyme tyrosinase. Compounds **6o, 6b, 6f,** and **6k** depicted excellent antityrosinase activity. Compound **6k** has the highest tyrosinase inhibition activity with an IC_50_ value of 5.34 ± 0.58 µM, which is as potent as the standard kojic acid (IC_50_ 6.04 ± 0.11 µM). From the structure–activity relationship, it could be inferred that the existence of pyridine nuclei and activating groups at the ortho and para positions of the benzylideneimine moiety is the main factor for tyrosinase activity. The presence of a methoxy substituent at the para position of benzylideneimine was the main cause of the outstanding activity of ligand **6k.** The molecular docking studies depicted that the conjugates **6 (a–o)** showed a much higher docking score than the standard kojic acid. The compound **6k** has an excellent binding affinity with a score of 8,999 and an ACE value of −219.66 kcal/mol. Three other compounds, **6o** (6.03 ± 0.95 µM), **6b (**7.22 ± 0.78 µM), and **6f** (7.97 ± 0.68 µM), also have remarkable activity. Furthermore, high binding affinities toward tyrosinase were exhibited by the compounds **6o** (IC_50_ 6.03 ± 0.95 µM), **6b** (IC_50_ 7.22 ± 0.78 µM), and **6f** (IC_50_ 7.97 ± 0.68 µM) with binding affinity scores of 8,908, 8,894, and 8,758 and ACE values of −152.73, −156.85, and −146.96 kcal/mol, respectively. Compounds **6o** and **6f** have two hydrogen bonding interactions with tyrosinase pocket amino acids. Compound **6b**, which does not have benzylideneimine in its structure, gives excellent activity due to the presence of two pyridine rings.

## Data Availability

The raw data supporting the conclusion of this article will be made available by the authors, without undue reservation.
