# Supplementary material for: Design and synthesis of novel dihydropyridine- and benzylideneimine-based tyrosinase inhibitors
Source: Front Pharmacol. 2024 Mar 26;15:1332184. doi: 10.3389/fphar.2024.1332184 (PMC11002185; doi:10.3389/fphar.2024.1332184)
Supplement: Supplementary file 1 [file DataSheet1.pdf]

Design and synthesis of novel dihydropyridine and benzylimine based tyrosinase inhibitors

Supporting Information File

All of the chemicals and solvents were bought from the Sigma Aldrich and Alfa Aesar and utilized directly for experimentation.The determination of melting points of the compounds wasdone by using Gallenkamp melting point apparatus via the open capillary method.FTIR spectra were recorded on Bruker alpha 2 FTIR specrophotometer with in spectral range of 4000–400cm^{-1}. Mass spectra were obtained using ESI LC TOF 6224 (Agilent), under positive ion mode. High resolution mass spectra were obtained on the 9.4 T Bruker FT-ICR-MS spectrometer in MeOH: MeCN (1: 1). TLC was determined on silica gel plates (Analtech 02521), solvent system EtOAc: hexanes (8: 2) and $CHCl_3$: CH_3OH (1: 1).

1. Purification of Amlodipine (4)

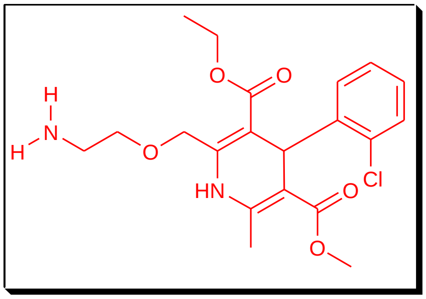

Amlodipine was purified by slurring in dichloromethane: water in 1:1 ratio followed by basification at 11 pH, extraction with dichloromethane solvent and purification of extract via preparative TLC. The purified drug was then analyzed for physical and chemical characterization. Purified Compound **4** was amorphous solid. The melting point noted was 200 °C and color was off white. On FTIR it showed characteristic peaks of primary amine *N*–H stretching at 3310.21, 3388.34 cm^{-1}Other band appeared at (ῡ cm^{-1}): 1682.48 (C=*O* Str. α, β–unsaturated ester), 1648.34 (*N–H* bend.secondary imine), 1600.65 (*N*-Hbend.Primary imine) 1472, 1429 (C=C str. aromatic ring), 1277 (C-*O* str. ether), 1201 (Ac-*O* str. Ester), 1096 (C-*O* str. ether), 1036 (C-*O* str. Of carbonyl) 757, 735 (C-*Cl*str.).

2. Synthesis of Compounds 6(a-o)

Amlodipine**(4)** and substituted aldehydes and ketones **6(a-o)** were refluxed using ethanol and catalytic amount of CH_3COOH for about 5-8 hours. The completion of reaction was observed by TLC. The solvent system was chloroform and methanol in ratio of (9.9:0.1). Then the reaction

mixture was quenched by aqueous $NaHCO_3$ solution (1 M). After it, the organic product was extracted using DCM (3 x 10 mL). Anhydrous $MgSO_4$ was used to dry the combined organic extract. Later, the solid product was filtered. After evaporation, preparative TLC was used for purification. Chloroform and methanol (90:10) were used as a solvent system. The purified organic product was dried over silica gel beads by keeping in desiccators for a week. Finally, the dried compound was collected for further analysis. (Mocanu&Cernatescu, 2008)

2.1. Synthesis of 3-ethyl 5-methyl-4-(2-chlorophenyl)-6-methyl-2-((2-methyleneamino)ethoxy)methyl)-1,4-dihydropyridine-3,5-dicarboxylate (6a)

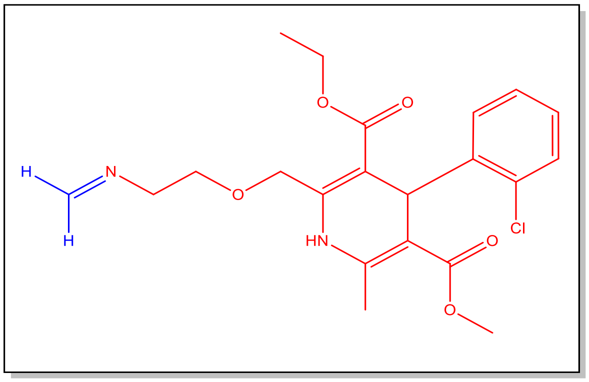

95% Yield:0.19 g, Color: Dark brown, Physical State: Semi solid, FTIR ῡ $(cm)^{-1}$: 2948.11 (C-H aromatic Str.), 1686.13 (C=*O* str. α, β-unsaturated ester), 1606.42 (C=*N* str. Imine), 1478.47, 1432.63 (C=C str. aromatic ring), 1278.5 (C-*O* str. ether), 1205.36 (Ac-*O* str. ester), 1093.53 (C-*O* str. ether), 732.39, 701.75 (C-*Cl* str.), MS-(ESI) *m/z*:$[M+H]^+$, 421.1, HRMS:Calcd. For $C_{21}H_{25}ClN_2O_5$, 421.1857 Found: 421.1850

2.2 Synthesis of 3–diethyl 3–dimethyl, 6,6' (*5Z,10E*)–2,14–dioxa–5,11–diaza pentadeca–5,10–diene–1,15–diyl)bis(4–(2–chlorophenyl))–2–methyl–1,4–dihydropyridine–3,5–dicarboxylate (6b)

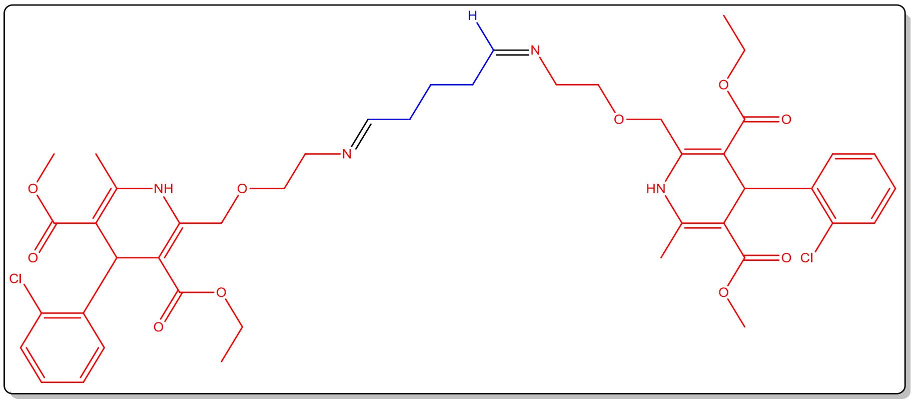

90% Yield:0.17 g, Color: Dark brown, Physical State: Semi solid, FTIR ῡ (cm)$^{-1}$: 2945.61 (C–H aromatic str.), 1685.48 (C=*O* str. α, β–unsaturated ester), 1605.41 (C=*N* str.), 1478.67, 1431.97 (C=C str. aromatic ring), 1278.47 (C-*O* str. ether)), 1203.84 (Ac–*O* str. ester), 1092.48 (C–*O* str. ether), 752.53, 702.61 (C–*Cl*str.).MS-(ESI) *m/z*: $[M+H]^{+}$, 881.3, HRMS:Calcd. For $C_{45}H_{54}Cl_2N_4O_{10},$ 881.3857Found: 881.3850.

2.3. Synthesis of 3–ethyl 5–methyl 4–(2–chlorophenyl)–6–methyl–2–((2–(((1 *E*, 2*E*)–3–phenylallylidene)amino)ethoxy)methyl)–1,4–dihydropyridine–3,5–dicarboxylate (6c)

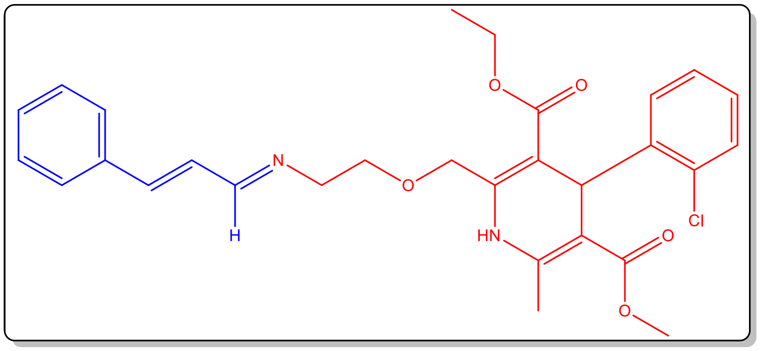

92% Yield:0.18 g, Color: Dark brown, Physical State: Semi solid, FTIR ῡ (cm)$^{-1}$: 2947.84 (C–H aromatic str.) 1680.02 (C=*O* str. α, β–unsaturated), 1602 (C=*N* str.), 1480.16, 1433 (C=C str. aromatic ring), 1265.6 (C-*O* str. ether)**,** 1206.3 (Ac–*O* str. ester), 1096.67 (C–*O* str. ether), 732.37, 698.93 (C–*Cl*str.) MS-(ESI) *m/z*:$[M+H]^{+}$, 523.1, HRMS: Calcd. For $C_{29}H_{31}ClN_2O_5,$ 523.1857 Found: 523.1850

2.4. Synthesis of 3–Ethyl 5–methyl (*E*)–2–((2–(benzylideneamino)ethoxy) methyl)–4–(2–chlorophenyl)–6–methyl–1,4–dihydropyridine–3,5–dicarboxylate (6d)

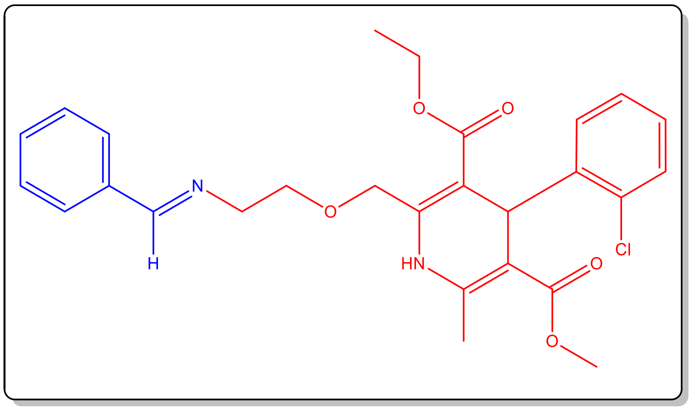

95% Yield:0.19 g, Color: Dark brown, Physical State: Semi solid, FTIR ʋ̄ (cm)$^{-1}$: 1687.56 (C=*O* str. α, β–unsaturated Ester), 1608 (C=*N* str. imine), 1480.23, 1433.76 (C=C str. aromatic ring), 1264.76 (C-*O* str. ether), 1208.52 (Ac–*O* str. ester), 1099.15 (C–*O* str. ether), 731.58, 700.9 (C–*Cl*str.), MS-(ESI) *m/z*:[M+H]$^+$, 497.1, HRMS:Calcd. For $C_{27}H_{29}ClN_2O_5$, 497.1857 Found: 497.1850

2.5. Synthesis of 3–ethyl 5–methyl (*E*)–2–((2–((2–chlorobenzylidene)amino) ethoxy)methyl)–4–((2–chlorophenyl)–6–methyl)–1,4–dihydropyridine–3,5–dicarboxylate (6e)

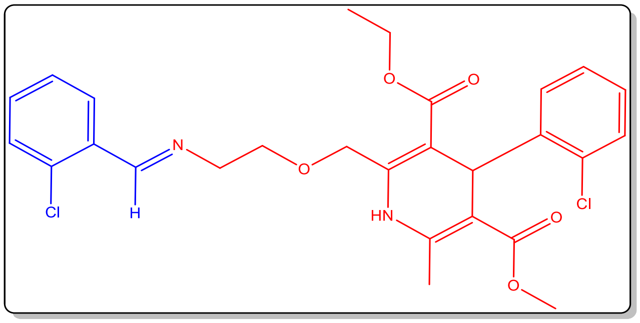

95% Yield:0.19 g, Color: Dark brown, Physical State: Semi solid, FTIR ʋ̄ (cm)$^{-1}$:2947.41 (C–H aromatic str.) 1687.22 (C=*O* str. α, β–unsaturated ester), 1645.52 (C=*N* str. imine), 1477.81, 1432.67 (C=C str. aromatic ring), 1279.14 (C-*O* str. ether), 1206.21 (Ac–*O* str. ester), 1089.2 (C–*O* str. ether), 734.22, 702.55 (C–*Cl*str.) MS-(ESI) *m/z*: [M+H]$^+$, 518.1, HRMS: Calcd. For $C_{26}H_{27}Cl_2N_2O_5$, 518.1857 Found: 518.1850

2.6. Synthesis of 3–ethyl 5–methyl (*E*)–2–((2–(4–bromobenzylidene)amino)ethoxy)methyl–4–((2–chlorophenyl)–6–methyl–1,4–dihydropyridine–3,5–dicarboxylate (6f)

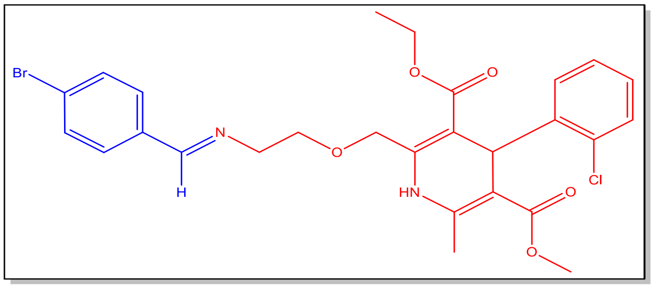

95% Yield:0.19 g, Color: Dark brown, Physical State: Semi solid, FTIR ῡ $(cm)^{-1}$:2949.53 (C–H aromatic str.) 1687.44 (C=*O* str. α, β–unsaturated ester), 1604.48 (C=*N* str. imine), 1522.08, 1479.67, 1433.77 (C=C str. aromatic ring), 1345.53 (C–*N* str. aromatic secondary amine) 1264.79 (C-*O* str. ether), 1208.75 (Ac–*O* str. ester), 1098.8 (C–*O* str. ether), 731.43, 701.57 (C–*Cl* str.), 600 (C–*Br* str.), MS-(ESI) *m/z*: $[M+H]^+$, 575.0, HRMS:Calcd. For $C_{27}H_{28}BrClN_2O_5$, 575.0857 Found: 575.0850.

2.7. Synthesis of 3–ethyl 5–methyl (*E*)–4–(2–chlorophenyl)–2–((2–((2–hydroxybenzylidene)amino)ethoxy)methyl)–6–methyl–1,4–dihydropyridine–3,5–dicarboxylate (6g)

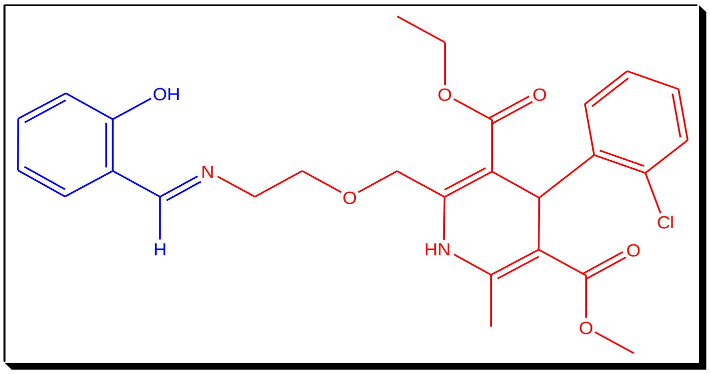

90% Yield:0.17 g, Color: Dark brown, Physical State: Semi solid, FTIR ῡ $(cm)^{-1}$: 3367 (*O*H str.), 2948.55 (C–H aromatic str.) 1686.46 (C=*O* str. α, β–unsaturated ester), 1606 (C=*N* str.), 1481.44, 1432.75 (C=C str. aromatic ring), 1281.12 (C-*O* str. ether), 1205.62 (Ac–*O* str. ester), 1094.14 (C–O str. ether), 754.8, 735.37 (C–*Cl*), MS-(ESI) *m/z*: $[M+H]^+$, 513.1, HRMS:Calcd. For $C_{27}H_{29}ClN_2O_6$, 513.1857 Found: 513.1850

2.8. Synthesis of 3–ethyl 5–methyl (*E*)–4–(2–chlorophenyl)–2–((2–((3–hydroxybenzylidene)amino)ethoxy)methyl)–6–methyl–1,4–dihydropyridine–3,5–dicarboxylate (6h)

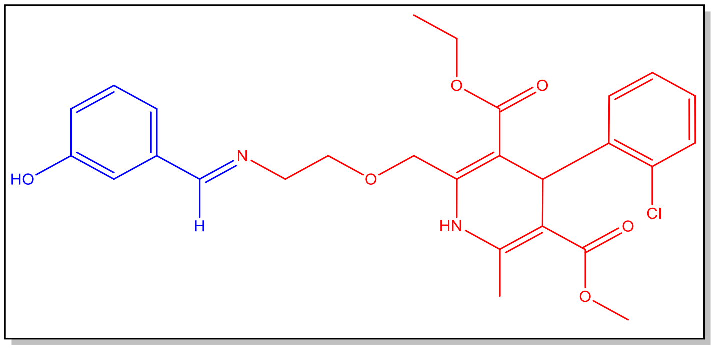

95% Yield:0.19 g, Color: Dark brown, Physical State: Semi solid, FTIR ῡ (cm)$^{-1}$:3396 (*O*H str.), 2950.91 (C–H aromatic str.) 1686.79 (C=*O* str. α, β–unsaturated), 1600.6 (C=*N* str.), 1479.81, 1433 (C=C str. aromatic ring), 1264.69 (C-*O*str. ether)**,** 1209.85 (Ac–*O* str. ether), 1098.51 (C–*O* str. ether), 731.93, 701.60 (C–*Cl*str.), MS-(ESI) *m/z*: [M+H]$^{+}$, 513.1, HRMS:Calcd. For $C_{27}H_{29}ClN_2O_6$, 513.1857 Found: 513.1850

2.9. Synthesis of 3–ethyl 5–methyl (*E*)–4–(2–chlorophenyl)–2–((2–((4– hydroxyl benzylidene)amino)ethoxy)methyl)–6–methyl –1,4–dihydropyridine–3,5–dicarboxylate (6i)

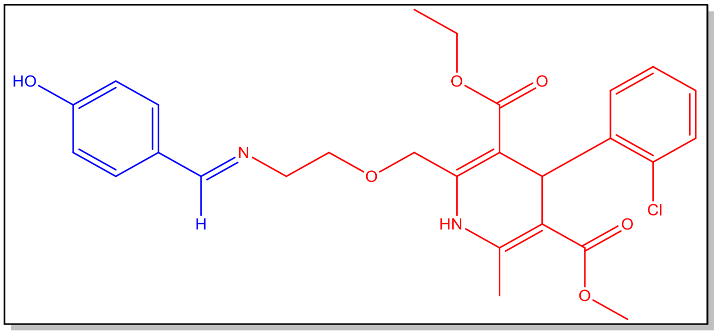

92% Yield:0.18 g, Color: Dark brown, Physical State: Semi solid, FTIR ῡ (cm)$^{-1}$: 3357 (*O*–H str.), 2950.87 (C–H aromatic str.) 1687.28 (C=*O* str. α, β–unsaturated ester), 1610.11 (C=*N* str.), 1513.85, 1481.68, 1433.97 (C=C str. aromatic ring), 1264.50 (C-O str. ether), 1209.95 (Ac–*O* str. ester), 1098.72 (C–*O* str. ether), 731.58, 701.95 (C–*Cl*str.), MS-(ESI) *m/z*: [M+H]$^{+}$, 513.1, HRMS:Calcd. For $C_{27}H_{29}ClN_2O_6$, 513.1857 Found: 513.1850

2.10. Synthesis of 3–ethyl 5–methyl (*E*)–4–(2–chlorophenyl)–2–((2–((2–hydroxy–3–methoxybenzylidene)amino)ethoxy)methyl)–6–methyl–1,4–dihydropyridine–3,5–dicar0boxylate (6j)

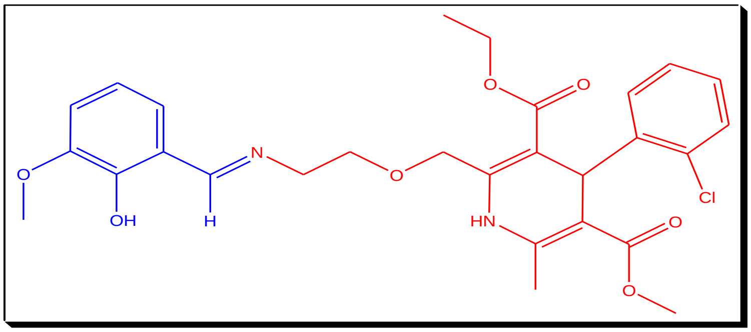

92% Yield:0.18 g, Color: Dark brown, Physical State: Semi solid, FTIR ῡ (cm)$^{-1}$: 3326 (*O*H str.), 2946.42 (C–H aromatic str.) 1687.63 (C=*O* str. α, β–unsaturated), 1633.19 (C=*N* str.), 1468.69, 1432.24 (C=C str. aromatic ring), 1276.53 (C-*O* str. ether), 1204.31 (Ac–*O* str. ester), 1092.03 (C–*O* str. ether), 755.49, 734.12 (C–*Cl*str.), MS-(ESI) *m/z*: [M+H]$^+$, 543.1, HRMS: Calcd. For $C_{28}H_{31}ClN_2O_7$, 543.1857 Found: 543.1850

2.11. Synthesis of 3–ethyl 5–methyl (*E*)–4–(2–chlorophenyl)–2–((2–((3,4–dimethoxybenzylidene)amino)ethoxy)methyl)–6–methyl–1,4–dihydropyridine–3,5–dicarboxylate (6k)

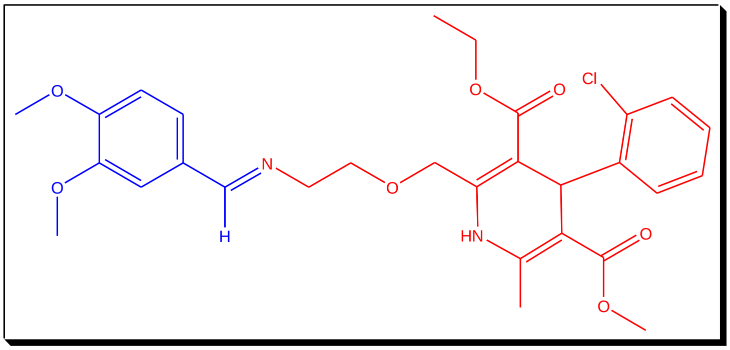

90% Yield:0.17 g, Color: Dark brown, Physical State: Semi solid, FTIR ῡ (cm)$^{-1}$:2950.35 (C–H aromatic str.) 1687.5 (C=*O* str.α, β–unsaturated ester), 1660 (C=*N* str. imine), 1514.73, 1466.34, 1433 (C=C str. aromatic ring), 1264.41 (C-*O* str. ether) 1208.1 (Ac–*O* str. ester), 1097.62 (C–*O* str. ether), 731.2, 701.25 (C–*Cl* str.), MS-(ESI) *m/z*: [M+H]$^+$, 557.2, HRMS:Calcd. For $C_{29}H_{33}ClN_2O_7$, 557.2857 Found: 557.2850

2.12. Synthesis of 3–ethyl 5–methyl (*Z*)–4–(2–chlorophenyl)–2–((2–((2–hydroxy–1,2–diphenylethyldiene)amino)ethoxy)methyl)–6–methyl–1,4–dihydro pyridine–3,5–dicarboxylate (6l)

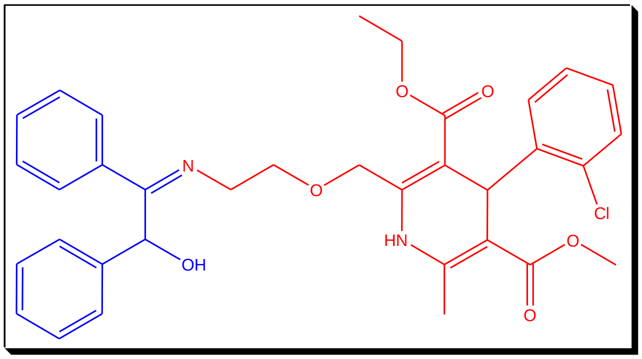

92% Yield:0.18 g, Color: Dark brown, Physical State: Semi solid, FTIR ῡ $(cm)^{-1}$: 3369.45 (*O*–H str.), 2946.9 (C–H aromatic str.) 1687.58 (C=*O* α, β–unsaturated ester), 1643.99 (C=*N* str. imine), 1476.02, 1432.3 (C=C str. aromatic ring), 1280.83 (C-O str. ether) 1204.48 (Ac–*O* str.), 1093.89 (C–*O* str. ether), 735.67, 702.51 (C–*Cl*str.) MS-(ESI) *m/z*: $[M+H]^+$, 603.2, HRMS:Calcd. For $C_{34}H_{35}ClN_2O_6$, 603.2857 Found: 603.2850

2.13. Synthesis of 3–ethyl 5–methyl (*E*)–4–(2–chlorophenyl)–6–methyl–2–((2–((1–((3–nitrophenyl)ethylidene)amino)ethoxy)methyl)–1,4–dihydropyridine–3,5–dicarboxylate (6m)

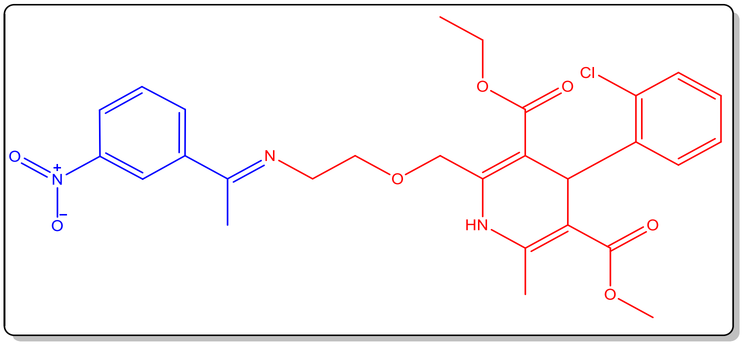

95% Yield:0.19 g, Color: Dark brown, Physical State: Semi solid, FTIR ῡ $(cm)^{-1}$: 2929.25 (C–H aromatic str.) 1727.29, 1683.14 (C=*O* α, β–unsaturated str.), 1595.67 (C=*N*), 1557 (*N*-*O* str. aromatic Nitro), 1478.94, 1433.27 (C=C str. aromatic ring), 1366 (*N*-*O* aromatic Nitro), 1280.75 (C-*O*str. ether), 1206.16 (Ac–*O* str. ester), 1094.7 (C–*O* str. ether), 754.83, 716.65 (C–*Cl*str.), MS-(ESI) *m/z*: $[M+H]^+$, 556.1, HRMS:Calcd. For $C_{28}H_{30}ClN_3O_7$, 556.1857 Found: 556.1850

2.14. Synthesis of 3–ethyl 5–methyl (*E*)–4–(2–chlorophenyl)–6–methyl–2–((2–((4–nitrobenzylidene)amino)ethoxy)methyl)–1,4–dihydropyridine–3,5–dicarboxylate (6n)

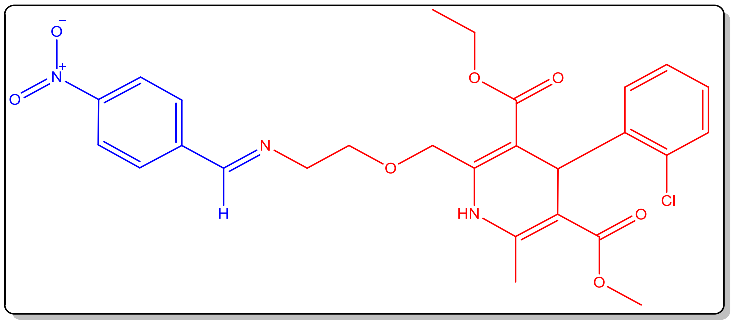

95% Yield:0.19 g, Color: Dark brown, Physical State: Semi solid, FTIR ῡ $(cm)^{-1}$:2949.17 (C–H str. aromatic), 1687.04 (C=*O* str. α,β–unsaturated), 1618.71 (C=*N* str. imine), 1483.95, 1433.19 (C=C str. aromatic ring), 1366 (*N-O* str. NO_2) 1265.27 (C-Ostr. ether) 1207.8 (Ac–O str. ester), 1097.2 (C–O str. ether), 821.33 (C–*N* str. aromatic NO_2) 732.46, 702.15 (C–*Cl*str.), MS-(ESI) *m/z*: $[M+H]^+$, 542.1, HRMS:Calcd. For $C_{27}H_{28}ClN_3O_7$, 542.1857 Found: 542.1850

2.15. Synthesis of 3–ethyl 5–methyl(*E*)–4–((2–((2–chlorophenyl)–2–((2–((2–hydroxy–5–nitrobenzylidene)amino)ethoxy)methyl)–6–methyl–1,4–dihydro pyridine–3,5–dicarboxylate (6o)

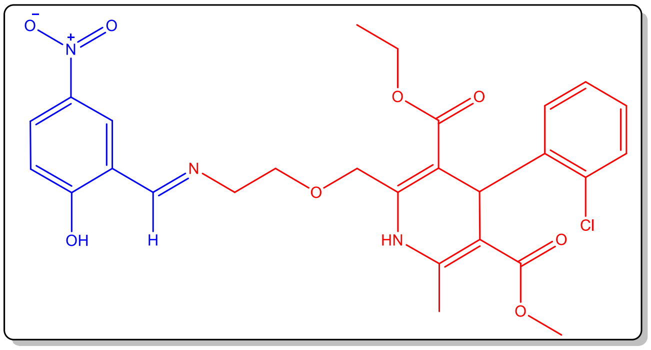

90% Yield:0.17 g, Color: Dark brown, Physical State: Semi solid, FTIR ῡ $(cm)^{-1}$:3386 (*O*H Str.), 2928.92 (C–H str. aromatic), 1687.48 (C=*O* str. α, β–unsaturated ester), 1644.11 (C=*N* str. imine), 1512.78, 1346.21 (str. Nitro), 1467.54, 1431.88 (C=C str. aromatic ring), 1273.38 (C-O str. ether), 1207 (Ac–*O* str. ester), 1096.57 (C–*O* str. ether), 834.17 (C–*N* str. Nitro), 734.89, 703.50 (C–*Cl* str.), MS-(ESI) *m/z*: $[M+H]^+$, 558.1, HRMS:Calcd. For $C_{27}H_{28}ClN_3O_8$, 558.1857 Found: 558.1850.

3. Tables

Table 1. Docking of Compounds **6 (a – o)** to Tyrosinase Protein (PDB ID: 2y9x) using *Patch Dock.*

**Ligand**	**[a]Score**	**[a](ACE) (kcal/mol)**	**Amino Acids Exhibit Potential For**			
			**Hydrogen Bond Interactions**	**[a]Distance (Å)**	**Hydrophobic Interactions**	**Pi–alkyl Interactions**
**6a**	5916	–189.09	–	–	Leu^{11}, Asp^{10}, Glu^{239}, Asn^{243}, Gly^{245}, Gly^{86}, Arg^{321}, Val^{88}, Gly^{47}, Ser^{26}, Leu^{240}	His^{251}
**6b**	8894	–156.85	Ser^{95}, Asp^{336}	2.05, 1.93	Trp^{72}, His^{56}, Thr^{334}, Tyr^{58}, Lys^{376}, Leu^{59}, Asp^{348}, Pro^{338}, Glu^{377}, Tyr^{62}, Ile^{96}, Arg^{108}, Pro^{110}, Tyr^{311}, Gln^{351}, Ser^{337}, Asp^{312}, Glu^{335}, Hoh^{2005}, Glu^{335}, Tyr^{314}, Trp^{238}, Ser^{316}, Glu^{317}	–
**6c**	6796	–197.41	–	–	Leu^{59}, Asn^{57}, His^{56}, Thr^{334}, Tyr^{314}, Asp^{336}, Glu^{356}, Asp^{358}, Glu^{335}, Val^{315}, Ser^{316},Trp^{238}, Trp^{72}, Phi^{87}	Val^{313},Tyr^{58}
**6d**	6606	–157.12	Gln^{74}, Tyr^{62}	2.75, 3.10	Tyr^{78}, Leu^{327}, Met^{325}, Phe^{105}, Thr^{324}, Ile^{328}, Lys^{70}, Tyr^{343}, Pro^{349}, Ile96^{438}, Glu^{377}, Lys^{93}, Ser^{95}, Glu^{97}, Gly^{61}, Asn^{57}	Pro^{110}

**6e**	6850	–237.56	Arg^{321}, Arg^{321}	3.19, 3.12	Ala^{250}, Gly^{245}, Gly^{47}, Pro^{46}, Pro^{12}, Glu^{239}, Leu^{11}, Asp^{10}	Ala^{27}, His^{251}, Ala^{246}, Leu^{9}
**6f**	8758	–146.96	Gln^{74}, Tyr^{62}	2.83, 3.21	Tyr^{343}, Glu^{340}, Asn^{57}, Pro^{349}, Gly^{61}, Lys^{93}, Ile^{96}, Glu^{377}, Tyr^{98}, Phe^{105}, Tyr^{78}, Thr^{324}, Leu^{327}, Lys^{70}	Pro^{110}
**6g**	6540	183.60	Tyr62	1.85	Phe^{87}, Trp^{72}, Trp^{238}, Asn^{57}, His^{57}, Thr^{334}, Val^{313}, Asp^{353}, Glu^{335}, Glu^{356}, Tyr^{314}, Val^{315}, Ser^{316}	Tyr^{58}
**6h**	6636	–183.82	–	–	Tyr^{314}, Val^{315}, Ser^{85}, Glu^{86}, Phe^{87}, Trp^{72}, $Thr2^{37}$, Trp^{238}, Asn^{57}, His^{56}, Thr^{334}, Val^{313}, Asp^{353}, Glu^{335}, Glu^{356}	Tyr^{58}, Leu^{9}
**6i**	6434	–195.74	–	–	Trp^{238}, Ser^{316}, Val^{315}, Tyr^{314}, Glu^{335}, Val^{313}, Asp^{312}, Glu^{317}, Thr^{334}, His^{56}, Asn^{57}, Tyr^{236}, Tyr^{58}, Thr^{237}, Trp^{72}, Phe^{37}	Leu^{9}
**6j**	6940	254.07	Met^{319},	3.24, 1.91	Trp^{72}, Phe^{65}, Val^{88}, Thr^{87}, Asn^{243}, Arg^{321}, Pro^{12}, Asp^{10}, His^{251}, Glu^{239}	Leu^{240}, Leu^{9}, Leu^{11}
**6k**	8999	–219.66	–	–	Ser^{316}, Trp^{238}, Thr^{237}, Phe^{37}, Trp^{72}, Leu^{240}, Tyr^{58}, Val^{313}, Glu^{317}, His^{56}, Thr^{334}, Asn^{57}, Asp^{312}, Tyr^{314}	Leu^{9}
**6l**	7226	–270.82	Arg^{321}	2.67	Leu^{240}, Pro^{46},	Ala^{27},Leu^{9},L

					Pro^{12}, Leu^{11}, Ser^{26}, Thr^{28}, Ser^{69}, Gly^{68}, Glu^{317}, Gly^{318}, Met ^{319} , Val^{88}, Thr^{87}, Asn^{243}, Gly^{245}	eu^{9},Ala^{246},Ala^{250},His^{251}
**6m**	6766	–174.14	Val^{315}, Tyr^{314}	3.40, 3.34	His^{56}, Asp^{336}, Thr^{334}, Glu^{335}, Trp^{238}, Val^{311}, Asp^{312}, Asp^{336}, Thr^{334}, Glu^{215}, Tyr^{314}, Trp^{238}, Asp^{312}, Leu^{39}	Tyr^{58}
**6n**	6646	–169.28	Val^{315}, Val^{313}	3.37, 1.95	Trp^{238}, Tyr^{314}, Ser^{316}, Glu^{335}, Tyr^{314}, Trp^{238}, His^{56}, Asp^{336}, Glu^{335}, Hoh^{2003}, Asp^{336}	Tyr^{58},Val^{313}
**6o**	8908	–152.73	Val^{315}	1.90	Trp^{238}, Ser^{316}, Ser^{316}, Glu^{317}, HOH^{2005}, Tyr^{314}, Leu^{59}, Trp^{238}, Asp^{312}, His^{56}, Thr^{334}, Glu^{335}	Tyr^{58}
**KojicAcid (Std.)**	2530	–68.92	Tyr^{128}	3.35	Leu^{9}, Asn^{70}, Phe^{65}, Trp^{72}, Phe^{87}, Thr^{237}, Leu^{240}	–

Calculated from a docked pose using the ligand interaction tool of *Patch Dock.*
